# *Vibrio cholerae* derived outer membrane vesicles modulate the inflammatory response of human intestinal epithelial cells by inducing microRNA-146a

**DOI:** 10.1038/s41598-019-43691-9

**Published:** 2019-05-10

**Authors:** Aziz Bitar, Kyaw Min Aung, Sun Nyunt Wai, Marie-Louise Hammarström

**Affiliations:** 1 Department of Clinical Microbiology, Infection and Immunology, Umeå University, Umeå, Sweden; 2 Department of Molecular Biology, the Laboratory for Molecular Infection Medicine, Umeå University, Umeå, Sweden

**Keywords:** Chemokines, Inflammasome, Mucosal immunology, Bacterial immune evasion, Pathogens

## Abstract

The small intestinal epithelium of *Vibrio cholerae* infected patients expresses the immunomodulatory microRNAs miR-146a and miR-155 at acute stage of disease. *V*. *cholerae* release outer membrane vesicles (OMVs) that serve as vehicles for translocation of virulence factors including *V*. *cholerae* cytolysin (VCC). The aim was to investigate whether OMVs, with and/or without VCC-cargo could be responsible for induction of microRNAs in intestinal epithelial cells and thereby contribute to immunomodulation. Polarized tight monolayers of T84 cells were challenged with OMVs of wildtype and a VCC deletion mutant of the non-O1/non-O139 (NOVC) *V*. *cholerae* strain V:5/04 and with soluble VCC. OMVs, with and without VCC-cargo, caused significantly increased levels of miR-146a. Increase was seen already after 2 hours challenge with OMVs and persisted after 12 hours. Challenge with soluble VCC caused significant increases in interleukin-8 (IL-8), tumour necrosis factor-α (TNF-α), CCL20, IL-1β, and IRAK2 mRNA levels while challenge with OMVs did not cause increases in expression levels of any of these mRNAs. These results suggest that *V*. *cholerae* bacteria release OMVs that induce miR-146a in order to pave the way for colonization by reducing the strength of an epithelial innate immune defence reaction and also preventing inflammation in the mucosa that factors like VCC can evoke.

## Introduction

*Vibrio cholerae* is the causal bacterium of the diarrheal disease cholera, a major health threat in developing and underdeveloped regions of the world and in areas struck by natural disasters such as floods^1^. It is a motile, Gram-negative bacterium acquired by intake of contaminated food or water. The diarrhoea is mainly attributed to the virulence factor cholera toxin (CT) and colonization of the small intestine attributed to the toxin-coregulated pilus (TCP)^2^. CT and TCP are expressed by the O1 and O139 serogroups of *V*. *cholerae*, which are the only serogroups known to cause epidemic cholera outbreaks^3^. There are also serotypes, collectively termed the *V*. *cholerae* non-O1, non-O139 (NOVC) strains, which lack the pathogenicity island encoding CT and TCP. Nonetheless, NOVC strains are able to cause diarrhoea by infecting the small intestine of humans^4,5^. In addition, they can cause extra-intestinal infections such as septic wound infection, meningitis, cellulitis, otitis, and septicaemia^2,6,7^. Clinical NOVC isolates can express a variety of virulence factors of which three, the CT-repeats in toxin (RTX), hemagglutinin protease (HapA) and *V*. *cholerae* cytolysin (VCC, also named haemolysin, HlyA) are expressed in almost all *V*. *cholerae* serogroups and hence likely toxigenic factors^2,6,8^. Using pure, soluble VCC and culture supernatants of a VCC deletion mutant of the *V*. *cholerae* O1 strain C6706 we previously showed that VCC is the major released factor that induces an inflammatory response in intestinal epithelial cells^9^.

One feature of Gram-negative bacteria is their capacity to form spherical membrane-enclosed particles, so called outer-membrane vesicles (OMVs)^10^. OMVs are constantly being discharged from the bacterial surface, with a maximum discharge in the late-logarithmic and stationary phases^11,12^. Typically, they are 50–200 nm in diameter and the vesicle membrane is usually composed of lipopolysaccharide (LPS), glycerophospholipids, and outer membrane proteins. Enriched with biologically active proteins, toxins, and other virulence factors, OMVs are suggested to have a role in bacteria-bacteria and bacteria-host interactions^10,13–16^. OMVs of the *V*. *cholerae* NOVC strain V:5/04 were shown to carry VCC^17^. OMVs have been suggested as causative agents for damaging as well as protective effects in infectious diseases^18^.

MicroRNAs are non-coding, short (19–25 nucleotides), single stranded RNA molecules that regulate gene expression at the post-transcriptional level^19–21^. miR-146a and miR-155 are two microRNAs shown to be involved in regulation of the acute inflammatory response after pathogen recognition through Toll-like receptors (TLRs)^19–21^. Identified target genes for miR-146a are the cytoplasmic adapter molecules Interleukin(IL)-1 receptor-associated kinase 1 (IRAK1), IRAK2, and Tumour necrosis factor(TNF) receptor-associated factor 6 (TRAF6). All are signalling molecules in the Nuclear factor κ-light-chain-enhancer of activated B cells (NF-κB) activation pathway^22,23^. The microRNA miR-375 was shown to play an important role in innate immunity by promoting intestinal epithelial cells to differentiate into goblet cells the producers of the protective mucus layer facing the gut lumen^24^. More recently, miR-375 was shown also to promote differentiation of enteroendocrine cells^25^ and regulate proliferation of stem cells of the intestinal epithelium^26^. All three microRNAs were shown to be involved in Crohn’s colitis and ulcerative colitis, two chronic inflammatory conditions of the human intestine^27^. Previously, we reported the finding that miR-146a and miR-155 were expressed in the duodenal epithelium of cholera patients at acute but not convalescent stage of disease^28^. Furthermore, challenge with live *V*. *cholerae* bacteria caused increased expression levels of miR-155 in an *in vitro* model of human intestinal epithelium^28^.

Whether OMVs of enteropathogens can influence levels of these microRNAs in the human intestinal mucosa is still scarcely investigated, particularly little is known about the role of OMVs and these microRNAs in the innate immune response of the epithelial cells. Here we aimed to gain knowledge about the capacities of OMVs, with and without VCC, to cause and/or modulate innate immune defence reactions of intestinal epithelial cells in *V*. *cholerae* infection. Polarized tight monolayers of T84 cells were challenged with OMVs of wildtype and a VCC deletion mutant of the NOVC *V*. *cholerae* strain V:5/04 as well as pure, soluble VCC. Tight monolayers were analysed for: (1) changes in expression levels of microRNAs miR-146a, miR-155 and miR-375, (2) changes in expression levels of mRNAs for the chemokines IL-8 and CCL20, the pro-inflammatory cytokine TNF-α, the inflammasome cytokines IL-1β and IL-18 and the miR-146a target genes IRAK1, IRAK2, and TRAF6, (3) changes in permeability and (4) cell death. We found that OMVs, with or without VCC-cargo, are not inflammatory by themselves but instead might down-regulate inflammation by causing increased levels of the immunomodulatory microRNA miR-146a.

## Methods

### Bacterial strains and isolation of outer membrane vesicles (OMVs)

*V*. *cholerae* V:5/04 non-O1 non-O139 clinical isolate 2004 (Swedish Institute of Infectious Diseases, Solna, Sweden) and its VCC deletion mutant (V:5/04/*Δvcc*)^17^ were used. Strains were frozen and stored at −80 °C in Luria-Bertani (LB) broth containing 15% glycerol. OMVs from the wildtype (wt) and the *Δvcc* V:5/04 strains were isolated from the bacterial cultures as previously described^13^. Briefly, bacteria were inoculated into a 200 mL culture flask containing LB broth and incubated for 16 hours at 37 °C with shaking. Bacterial cells were removed from the culture fluid by centrifugation at 5000 × *g* for 30 minutes. The supernatant was sterile filtered through a 0.22-μm polyvinylidene difluoride (PVDF) membrane filter (Millipore, Merck Chemicals and Life Science, Solna, Sweden). The cell-free supernatant was centrifuged at 100,000 × *g* for 2 hours at 4 °C in a 45 Ti rotor (Beckman Instrument) to obtain the vesicles, *i*.*e*. crude preparation of OMVs (OMV-c). The OMV-c were suspended in Dulbecco’s modified Eagle’s medium (DMEM) and either used in tight monolayer challenge experiments or further purified by density gradient centrifugation as described^17^. Briefly, gradient of 0.4 mL 45%, 0.5 mL 35%, 0.6 mL 30%, 0.6 mL 25%, 0.6 mL 20%, 0.5 mL 15% and 0.6 mL 10% Optiprep (Sigma-Aldrich Sweden, Stockholm, Sweden) was made in a 4 mL ultracentrifugation tube. OMV-c samples were loaded on top of the gradient and centrifuged at 180,000 × *g* for 3 hours at 4 °C in a SW60Ti rotor (Beckman Instrument). After centrifugation, a 100–300 µl fraction was collected from the 35 to 30% border, washed in 50 mL 20 mM Tris-HCl buffer (pH 8.0) and centrifuged at 100,000 × *g* for 2 hours at 4 °C in a 45 Ti rotor (Beckman Instrument). Pellets with purified OMVs (OMV-p) were suspended in 500 µl DMEM for tight monolayer challenge experiments. Totally 3 wt-OMV-p preparations and 3 *Δvcc*-OMV-p preparations were utilized for challenge of polarized tight monolayers. Two of the wt-OMV-p preparations and 2 of the *Δvcc*-OMV-p preparations were compared to their respective OMV-c preparation in tight monolayer challenge experiments.

### Nanoparticle tracking analysis of OMV samples

Size and concentration of OMVs were estimated by nanoparticle tracking analysis using the ZetaView PMX 110 (Particle Metrix, Meerbusch, Germany) and the appurtenant software (ZetaView 8.02.28) according to manufacturer’s instructions^29^. Results are given as an average based on five independent measurements for each OMV sample.

### Transmission electron microscopy

OMV samples were placed on carbon-coated Formvar grids after negative staining with 0.1% uranyl acetate and examined under an electron microscope. Micrographs were taken with a JEOL 2000EX electron microscope (JEOL, Akishima, Japan) operated at an accelerating voltage of 100 kV.

### Sodium dodecyl sulphate-polyacrylamide gel electrophoresis (SDS-PAGE) and immunoblot analysis

The protein content of OMV-c and OMV-p samples were determined using the bicinchoninic acid (BCA) Bradford assay kit (Thermo Scientific Pierce, Rockford, IL, USA). Twenty µg per OMV-sample, was precipitated with 50% (w/v) trichloroacetic acid 1:4, incubated on ice for 15 minutes and subsequently centrifuged at 14,000 rpm for 15 minutes at 4 °C. The pellet was washed twice with ice-cold phosphate buffered saline (PBS; pH 7.2) and resuspended in 10 mM Tris-HCl buffer (pH 6.8) containing 10% glycerol, 0.05% bromphenol blue, 2% SDS, 5% 2-mercaptoethanol, and separated according to size by 13.5% SDS-PAGE with a discontinuous buffer system at a constant voltage of 60 V for the stacking gel and 120 V for the resolving gel. The proteins in the gel were transferred to a PVDF membrane (Millipore) in standard transfer buffer with a Bio-Rad semidry transfer system at 23 V for 35 min. After the transfer was completed, the membrane was blocked with PBS containing 5% skim milk and 0.05% Tween-20 at 4 °C overnight. The immunoblot membrane was incubated with a 1:10,000 dilution of polyclonal rabbit anti-VCC antiserum as primary antibody for 1 hour. Horseradish peroxidase conjugated goat anti-rabbit IgG antibody (AgriSera, Umeå, Sweden) was used as a secondary antibody at a final dilution of 1:25,000. The ECL+ chemiluminescence system (GE Healthcare Life Sciences) was used to detect immunoreactive bands that were recorded using a Fluor-S MultiImager (BioRad). Amount of VCC in OMV-p samples were estimated by comparing the density of bands revealed by anti-VCC immunobloting of OMV samples and recombinant purified VCC run in the same SDS-PAGE. Quantification of density was done using the Quantity One 1-D Analysis software.

### Culture of T84 cells, establishment and challenge of polarized tight monolayers and measurement of permeability

The colon carcinoma cell line T84 (American Type Culture Collection, Rockville, MD, USA) was cultured at 37 °C in a humidified atmosphere with 5% CO_2_. Tissue culture medium used was a 1:1 mixture of DMEM and Ham’s F12 medium with 15 mM HEPES buffer, 8% fetal calf serum, 2 mM L-glutamine, 100 units/mL penicillin and 85 µg/mL streptomycin. All tissue culture media components were from Invitrogen (Paisley, UK). Confluent cultures were trypsinized by incubation for 5 min at 37 °C with PBS (pH 7.2) containing 0.25% trypsin and 0.5% EDTA. The cells were allowed to recover for 60 minutes at 37 °C in complete culture medium after trypsinization. Tight monolayers were established by seeding 0.5 × 10^6^ T84 cells in 0.5 mL complete culture medium per well in Transwell inserts with semi-permeable polycarbonate membrane supports with 12 mm diameter and 0.4 μm pore size (Costar 3401; Corning Incorporated, Corning, NY, USA). Complete culture medium (1.5 mL) was also added to the lower chamber outside of the inserts. From the second day after seeding, medium was changed daily and transepithelial electrical resistance (TER) was measured by using the Millicell Electrical Resistance System (Millipore) with chopstick electrodes. A TER ≥1000 Ohm was considered an indicator of a polarized tight monolayer^30^. Challenge of monolayers was done by replacing medium in the upper chamber with 0.5 mL of OMV preparations or 0.5 mL medium with 160 ng VCC/mL (*i.e*. 80 ng VCC/monolayer) and incubated in a humidified atmosphere with 5% CO_2_ at 37 °C for 2, 5, and 12 hours. Challenge with OMV preparations and VCC was performed in quadruplicate with four sham-treated tight monolayers in the same tissue culture plate as controls. Permeability was estimated as TER. Results are first calculated as TER after incubation in % of TER before incubation in the same T84 monolayer well using the formula: %TER = TER_[after]_/TER_[before]_ × 100. In order to give possibility to compile results from independent experiments, the results for individual monolayers were thereafter normalized to the mean %TER of the relevant sham-treated controls and expressed as % of Control. All sham-treated T84 monolayers maintained stable TER values (97 ± 3.6%) after 5 hours incubation.

### Cytolysis assay

Culture medium was collected from the lower chamber of monolayer cultures at the end of incubation and cell death estimated as the lactate dehydrogenase (LDH) activity released using the Cytotoxicity Detection Kit (LDH) (Roche, Mannheim, Germany). Percent cytolysis was calculated as: [(experimental optical density (OD) at 405 nm value − low control OD_405_ value)/(high control OD_405_ value − low control OD_405_ value)] × 100. “Low control” is the spontaneous release of LDH from sham-treated tight monolayers and “high control” is the LDH activity in supernatants from parallel tight monolayers incubated in the presence of 2% Triton X100.

### RNA extraction

Membranes with OMV- and VCC-challenged, and sham-treated T84 tight monolayers were cut out, washed two times with RNase-free PBS and frozen in RTL-buffer (RNeasy Mini Kit; Qiagen, Sollentuna, Sweden) supplemented with 0.1 M 2-mercaptoetanol and stored at −80 °C until RNA extraction. Total RNA was extracted by using the RNeasy Mini Kit (Qiagen) according to the manufacturer’s instructions and dissolved in RNase-free water. RNase inhibitor, recombinant RNasin (1000 units/mL; Promega, Madison, WI, USA), was added to each sample and samples were stored at −80 °C until analysis of microRNA and mRNA expression levels.

### Determination of expression levels of microRNAs

Expression levels of microRNAs miR-146a, miR-155, and miR-375, and small nuclear RNA RNU48 were determined by real-time quantitative reverse transcriptase-polymerase chain reaction (qRT-PCR) using the TaqMan MiRNA Reverse Transcription (RT) Kit and TaqMan MiRNA (TM) Assay primers for human miR-146a (hsa miR-146a: 000468), miR-155 (hsa-miR-155: 002623), miR-375 (hsa-miR-375: 000564), and RNU48 (RNU48: 001006) (Life Technologies, Foster City, CA, USA). RNA concentration was determined by measuring OD at 260 nm in a NanoDrop 1000 spectrophotometer V3.0.0 (Saveen Werner, Limhamn, Sweden) and purity estimated as the OD_260_/OD_280_ ratio. Only samples with OD_260_/OD_280_ ratios above 1.95 were included. The samples were analysed in duplicates using the ABI prism 7900 HT sequence detection system (Applied Biosystems, Foster City, CA, USA). Each real-time qRT-PCR reaction contained 200 ng RNA. Expression levels were estimated by calculating the relative quantity (RQ) using the 2^(−∆∆ct)^-method^31^. The levels of microRNAs were normalized to RNU48 by calculating the Δct between the ct-value of the microRNA species of interest and the ct-value of RNU48 in the same sample. ΔΔct was thereafter calculated as the microRNA Δct-value in the OMV- or VCC challenged sample minus the median microRNA Δct-value of the four sham-treated control T84 monolayers of the respective challenge experiment.

### Determination of expression levels of mRNAs

Quantification of mRNAs for IRAK1, IRAK2, TRAF6, IL-1β, IL-18, CCL20, IL-8 and TNF-α was performed using real-time qRT-PCR assays based on the EZ-technology, with primers placed in different exons, a reporter dye marked probe placed over the exon boundary in the amplicon and using the 3′-primer as template for specific reverse transcription of mRNA in total RNA performed in each run as described^32^. Quantification of IRAK1, IRAK2, TRAF6, IL-1β, IL-18, and CCL20 mRNAs was performed using the Taqman gene Expression Assays Hs01018347_m1, Hs00176394_m1, Hs00377558_m1, Hs00367225_m1, Hs_01038788_m1, and Hs00355476_m1 (Applied Biosystems), respectively. qRT-PCR assays for IL-8 and TNF-α were constructed in the laboratory and include an RNA copy standard, for details see^30,32^. The concentration of 18S rRNA was determined in each sample using a commercial real-time qRT-PCR assay (Applied Biosystems) and expressed as arbitrary units from a standard curve of serial dilutions of a preparation of total RNA from human peripheral blood mononuclear cells^33^. One unit was defined as the amount of 18S rRNA in 1 pg of RNA and corresponds to approximately 1 epithelial cell^34^. All samples included in the study contained >40 units 18S rRNA per reaction mixture.

Samples were analysed in triplicates for mRNA and 18S rRNA concentrations using the ABI prism 7900 HT sequence detection system (Applied Biosystems). mRNA concentrations were normalized to the 18S rRNA concentration in the sample by calculating mRNA copies/18S rRNA unit for IL-8 and TNF-α mRNAs that were analysed by assays with an RNA copy standard and RQ calculated by the 2^(−∆∆ct)^-method, where ∆∆ct is ∆ct for the sample minus the median of the ∆ct-values of the four sham-treated control T84 tight monolayers of the respective experiment for mRNAs for which no copy standard was available.

### Statistical analyses

Statistical analysis of differences in TER, microRNA levels and mRNA levels in polarized tight monolayers challenged with OMVs compared to sham-treated monolayers, was performed using one-way ANOVA with Dunnett’s multiple comparison post-test. Analysis of differences in miR-146a levels after challenge with different doses of wt-OMV-p was performed using one-way ANOVA with post-test for linear trend. Pairwise comparison between monolayers challenged with OMV samples before and after density gradient centrifugation, monolayers challenged with wt-OMV-p for various length of time compared to monolayers sham-treated in parallel over the same time-period, and monolayers challenged with VCC protein compared to sham-treated monolayers was done using Student’s t-test. The Prism 7 computer program (GraphPad Software, San Diego, CA, USA) was used for the statistical analyses. Two-sided analysis was used throughout. A *P*-value < 0.05 was regarded as statistically significant. Descriptive values of TER, microRNA and mRNA expression levels are given as mean ± 1 standard deviation (SD). Descriptive values of results from nanoparticle tracking analysis of OMV samples are given as mean and standard error of the mean (SEM).

## Results

### Characterization of OMV preparations

**Figure 1:**
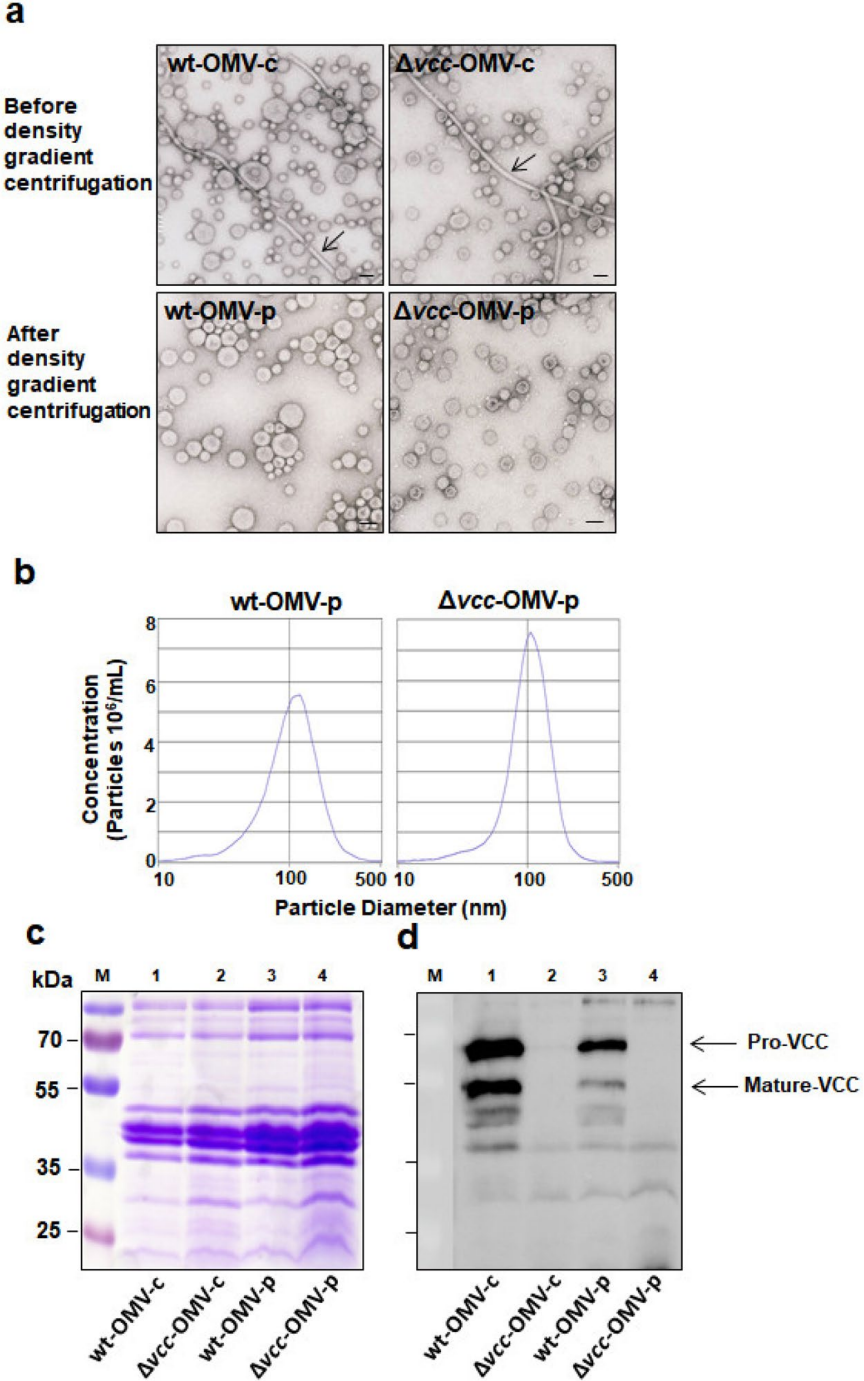
OMVs subjected to density gradient centrifugation exhibit high purity. (**a**) Transmission electron microscopy analysis of OMV preparations from wildtype *V*. *cholerae* V:5/04 (wt-OMV) and its VCC deletion mutant (Δ*vcc*-OMV) before (wt-OMV-c and Δ*vcc*-OMV-c) and after (wt-OMV-p and Δ*vcc*-OMV-p) density gradient centrifugation. Arrows indicate flagellin filaments. Bars in the lower right of micrographs indicate 100 nm. (**b**) Nanoparticle tracking analysis of density gradient centrifugation purified OMVs from wildtype *V*. *cholerae* V:5/04 (wt-OMV-p) and its VCC deletion mutant (Δ*vcc*-OMV-p). (**c**) Coomassie-brilliant-blue-stained SDS-PAGE gel and (**d**) immunoblot analysis of the same gel using polyclonal anti-VCC antiserum. Samples analysed: wt-OMV-c (lane 1), Δ*vcc*-OMV-c (lane 2), wt-OMV-p (lane 3) and Δ*vcc*-OMV-p (lane 4). Molecular weight markers were run in lane M. Molecular weights of the markers are given in kDa to the left of the gel in (**c**). Arrows in (**d**) indicate the position of the pro-VCC and the mature VCC protein, respectively. The full-length SDS-PAGE gel and its anti-VCC immunoblot are shown in Supplementary Fig. S1.

TEM analysis revealed OMVs as typical spherical vesicles with an approximate diameter of approximately 100 nm (Fig. 1a). OMV-c preparations contained a mixture of vesicles and non-vesicular components resembling flagellar filaments (Fig. 1a, upper row), while only vesicles were detected in the density gradient centrifugation purified OMV-p preparations (Fig. 1a, lower row). OMVs from wildtype bacteria seemed more heterogenous in size than OMVs from VCC deleted bacteria (Fig. 1a, left column compared to right column). The number of OMV particles relative to protein was generally lower in OMV-p from wildtype bacteria (mean ± SEM: 56 ± 22 × 10^6^ vesicles/mg protein; n = 3) compared to OMV-p preparation from bacteria with a deleted VCC gene (mean ± SEM: 129 ± 44 × 10^6^ vesicles/mg protein; n = 3). At the SDS-PAGE resolution level, OMV-samples showed similarity in major proteins (Fig. 1c and Supplementary Fig. S1a). SDS-PAGE followed by anti-VCC immunoblot analysis confirmed the presence of VCC in wt-OMVs and absence of VCC in *Δvcc*-OMVs, respectively (Fig. 1d, and Supplementary Figs S1a,b and S2a,b). VCC is synthesized in the bacteria in the form of an 82 kDa precursor polypeptide, designated pre-pro-VCC^35^. The soluble, recombinant VCC used in this study was mainly in this form (Supplementary Fig. S2a,b). During secretion through the bacterial membranes, the N-terminal signal peptide is removed generating a 79 kDa pro-VCC peptide from which the N-terminal pro-domain is proteolytically removed generating the functional mature form of VCC (68 kDa; ref.^36^). wt-OMVs carried the mature form of VCC (Supplementary Figs S1a,b and S2a,b) and one preparation also carried significant amounts of pro-VCC (Supplementary Fig. S2a,b), while the pre-pro-form was not detected in OMVs. OMVs also carried VCC polypeptides of approximately 55 kDa molecular weight (Supplementary Figs S1a,b and S2a,b). Such fragments can be generated from proteolytic cleavage possibly by *V*. *cholerae* PrtV^9^, an enzyme previously shown to be carried by OMVs^37^. Comparisons between intensities of bands from immunodetection with anti-VCC antiserum in wt-OMV-p preparations and dilutions of the recombinant, soluble VCC protein showed that VCC constitutes 0.097% of the total protein of the OMVs (Supplementary Fig. S2c).

### Purified *V*. *cholerae* OMVs induce further tightening of T84 tight monolayers

**Figure 2:**
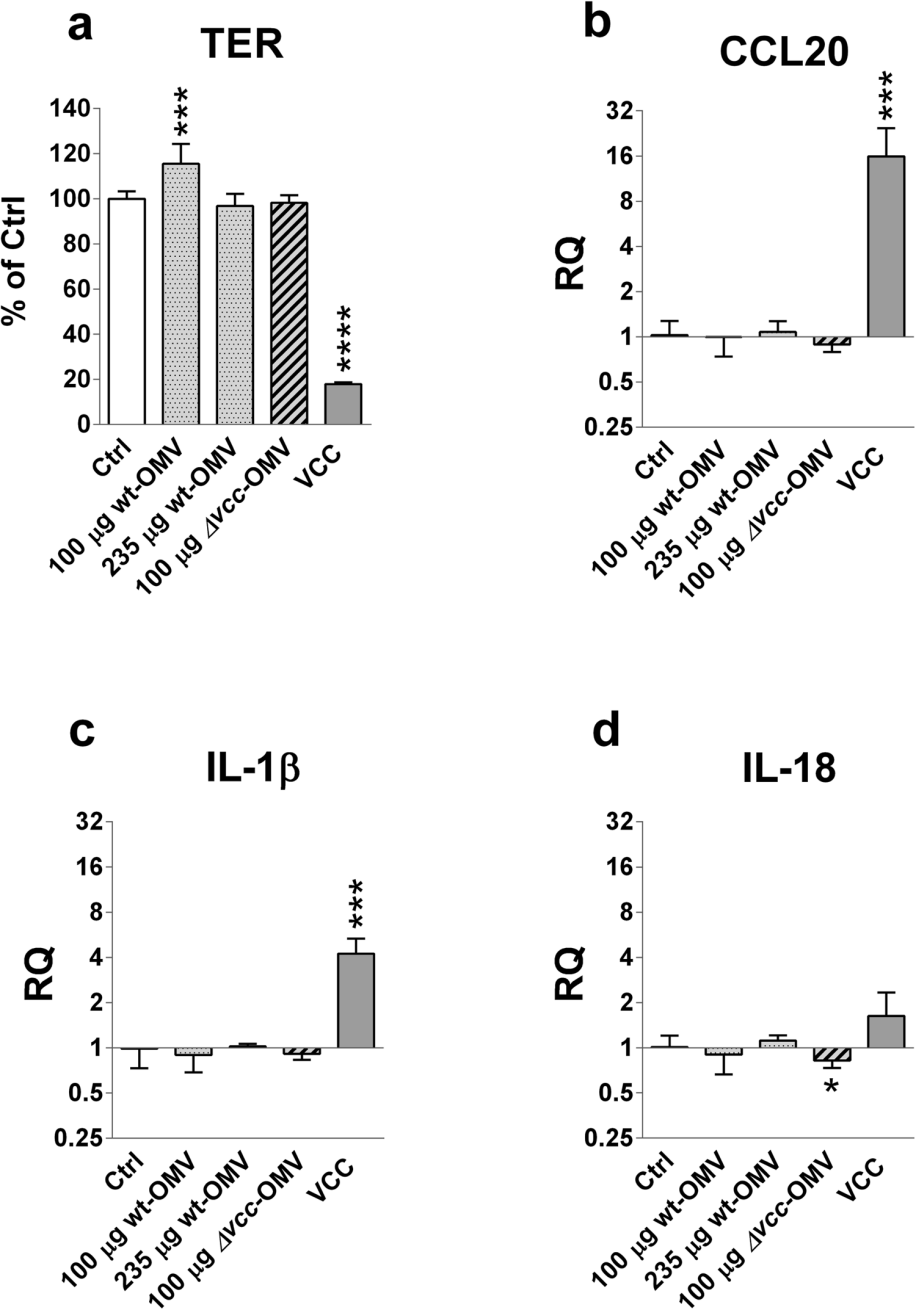
Soluble VCC causes decreased TER and increased expression levels of CCL20 and IL-1β mRNA in T84 polarized tight monolayer cells, while OMV-bound VCC causes increased TER and no change in cytokine mRNA levels. Polarized tight monolayers of T84 cells were challenged at the apical side with density gradient centrifugation purified OMVs from the *V*. *cholerae* strain V:5/04 (wt-OMV-p) and its VCC deletion mutant (Δ*vcc*-OMV-p). Monolayers were challenged with 100 µg wt-OMV-p/monolayer (100 µg wt-OMV, dotted bar; n = 12), 235 µg wt-OMV-p/monolayer (235 µg wt-OMV, dotted bar; n = 4), 100 µg Δ*vcc*-OMV-p/monolayer (100 µg Δ*vcc*-OMV, striped bar; n = 12) and 80 ng soluble VCC (VCC, grey bar; n = 4) or sham-treated monolayers (Ctrl, open bar; n = 16). (**a**) Changes in TER. (**b**–**d**) Changes in levels of mRNA for CCL20 (**b**), IL-1β (**c**) and IL-18 (**d**). Results in (**a**) are given as % of controls (% of Ctrl) for individual monolayers relative to the mean %TER of sham-treated control monolayers in the respective experiment. Results in (**b**–**d**) are given as relative quantity (RQ) compared to the median level of sham-treated control monolayers in the respective experiment. Challenges were done with the indicated amount of OMV protein/monolayer for 5 hours. Three batches of wt-OMV-p and Δ*vcc*-OMV-p, respectively, were tested in 3 independent experiments. n = number of monolayers. Bars indicate mean + 1 SD. Statistically significant differences are shown; ****P-value < 0.0001, ***P-value < 0.001, *P-value < 0.05.

The ability of *V*. *cholerae* derived OMVs to alter intestinal epithelial barrier function was assessed as changes in tight monolayer permeability measured as TER^38^. OMVs and soluble VCC were added apically to polarized tight monolayers of T84 cells, *i*.*e*. in the upper chamber of monolayers grown in Trans-well inserts, and TER was measured before and after challenge. Challenge with soluble VCC was done at an inflammatory but non-toxic contraction (160 ng VCC/mL; 80 ng/monolayer)^9^. In line with our previous study, challenge with soluble VCC caused a pronounced decrease in TER upon 5 hours challenge reaching below 20% of that in sham-treated control monolayers (Fig. 2a). In contrast, challenge with OMV-bound VCC did not cause decreases in TER. Thus, 5 hours challenge with 100 µg wt-OMVs/monolayer, *i*.*e*. approximately 97 ng VCC/monolayer, caused a rather unexpected significant increase in TER compared to sham-treated controls (Fig. 2a). This tightening effect was seen repeatedly with three different wt-OMV-p batches. None of three different batches of *Δvcc*-OMV-p caused significant changes in TER in monolayers challenged with the same amount of OMV-protein (100 µg/monolayer; Fig. 2a). When the amount of wt-OMVs was increased to 235 µg/monolayer, *i*.*e*. ≈228 ng VCC/monolayer, an increase in TER was no longer seen (Fig. 2a). This difference could be explained by the fact that the amount of VCC is toxic for the cells^9^. Alternatively, changes in TER might be dependent on the number of OMVs the monolayer is exposed to. In the three experiments where an increase in TER was seen the challenge was done with 1.5–8.9 × 10^6^ wt-OMV-p vesicles/monolayer, while in the experiments where the TER was unchanged the monolayers were exposed to 20.9 × 10^6^ wt-OMV-p vesicles/monolayer or 9.7–21.6 × 10^6^ *Δvcc*-OMV-p vesicles/monolayer (Fig. 2a).

Only OMV-c preparations caused significant decrease in TER (Supplementary Fig. S3a). These effects disappeared after density gradient centrifugation of the respective sample (Supplementary Fig. S3a) suggesting that decreases in TER were caused by non-vesical associated materials in the OMV-c preparations.

None of the OMV preparations or VCC protein alone caused increased release of LDH above the levels released from sham-treated controls (<2% of maximum release).

### Purified *V*. *cholerae* OMVs are not inflammatory

Since purified *V*. *cholerae* OMVs were shown to positively alter epithelial integrity by increasing TER in tight monolayers we next investigated whether OMVs could modulate cytokine responses in intestinal epithelial cells in the T84 tight monolayer model. We have previously shown that culture supernatant from *V*. *cholerae* bacteria can cause increased expression levels of the granulocyte attracting chemokine IL-8, the pro-inflammatory cytokine TNF-α and the inflammasome cytokine IL-1β mRNAs in T84 tight monolayers^9,28^. Genome-wide hybridization bead array screening for gene expression showed that T84 monolayers also constitutively express detectable levels of mRNA for the lymphocyte attracting chemokine CCL20 and very high levels of the inflammasome cytokine IL-18^39^. Therefore, the five cytokines IL-8, TNF-α, CCL20, IL-1β and IL-18 were selected for investigation of to what extent their mRNA expression levels in T84 monolayer cells are influenced by challenge with OMVs. Significant constitutive expression of mRNAs for the five cytokines was confirmed in sham-treated control monolayers by qRT-PCR (summarized in Supplementary Table S1). In agreement with our previous study^9^, soluble VCC induced highly significant increases in mRNA expression levels for IL-8 and TNF-α (Supplementary Fig. S3e,f). Notably, challenge with soluble VCC also caused significant increases in CCL20 and IL-1β mRNA levels (Fig. 2b,c). In contrast, no increase in levels of mRNA for the cytokines was seen after challenge with OMV-bound VCC at either 97 ng/monolayer (100 µg wt-OMV-p protein/monolayer) or and 228 ng VCC/monolayer (235 µg wt-OMV-p protein/monolayer; Fig. 2b–d and Supplementary Fig. S3e,f). Likewise, purified preparations of OMVs without VCC-cargo (*Δvcc*-OMV-p) did not induce an inflammatory response in tight monolayer cells (Fig. 2b–d). Two of the three *Δvcc*-OMV-p samples even caused decreases in IL-18 and to some extent also CCL20 mRNA levels (Supplementary Fig. S3b,d).

Challenge with crude OMVs (OMV-c), with and without VCC-cargo, caused marginal increases in IL-8, TNF-α and IL-1β mRNA levels, but these effects disappeared after density gradient centrifugation suggesting that they are caused by non-vesicular components such as contaminating flagellin (Supplementary Fig. S3c,e,f).

### *V*. *cholerae* OMVs induce miR-146a

**Figure 3:**
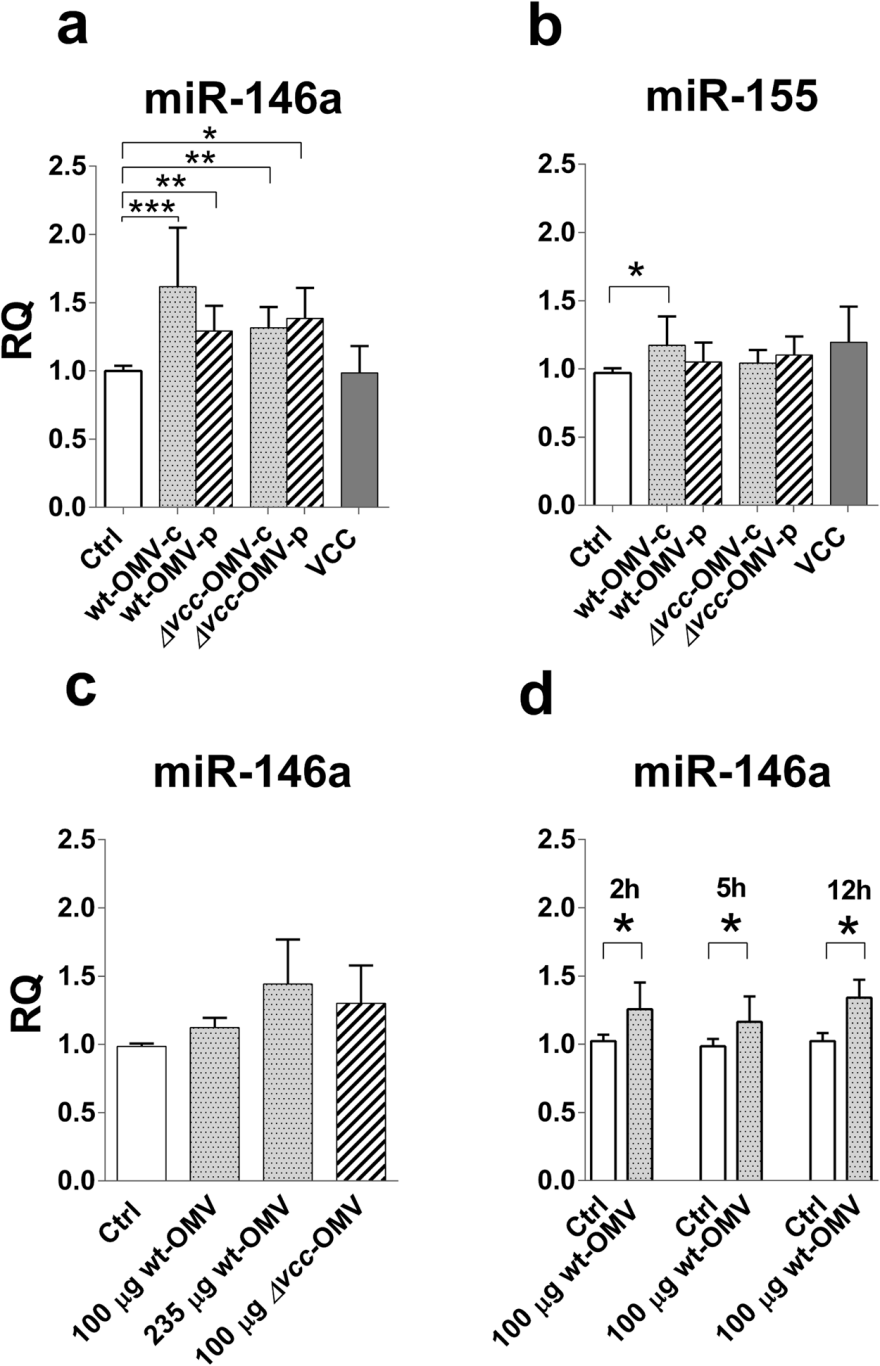
*V*. *cholerae* derived OMVs, but not VCC, induce increased expression levels of miR-146a in T84 polarized tight monolayer cells. (**a**,**b**) Polarized tight monolayers of T84 cells were challenged at the apical side with 100 µg OMV protein derived from the *V*. *cholerae* strain V:5/04 and its VCC deletion mutant before (wt-OMV-c and *Δvcc*-OMV-c) and after (wt-OMV-p and *Δvcc*-OMV-p) density gradient centrifugation and with 80 ng soluble VCC (VCC) for 5 hours. Challenged monolayers and sham-treated controls (Ctrl) were thereafter monitored for changes in miR-146a (**a**) and miR-155 (**b**) levels. n = 8 challenged monolayers for each OMV-type, 4 monolayers challenged with VCC and 12 sham-treated monolayers. (**c**) Expression levels of miR-146a in monolayers challenged with 100 µg (100 µg wt-OMV) and 235 µg (235 µg wt-OMV) of the same density gradient centrifugation purified OMV sample from wildtype bacteria of the *V*. *cholerae* strain V:5/04, and 100 µg of density gradient centrifugation purified OMV of the VCC deletion mutant for 5 hours and their sham-treated control monolayers (Ctrl). n = 4 challenged monolayers per dose and OMV-type and 4 sham-treated monolayers. (**d**) Expression levels of miR-146a in monolayers challenged with 100 µg of one density gradient centrifugation purified OMV sample from the wildtype *V*. *cholerae* strain V:5/04 (100 µg wt-OMV) for 2, 5 and 12 hours and their respective sham-treated control monolayers (Ctrl). n = 4 challenged and 4 sham-treated monolayers per time-point. (**a**–**d**) Amounts of miR-146a were determined by real-time qRT-PCR and normalized to the content of RNU48 in the sample. Results are expressed as relative quantity (RQ) relative to the median of sham-treated tight monolayers incubated in parallel with monolayers challenged with OMV or VCC. Bars indicate mean RQ + 1 SD. Statistically significant differences are shown, ***P-value < 0.001, **P-value < 0.01 and *P-value < 0.05.

We recently reported that live *V*. *cholerae* bacteria can induce microRNAs, particularly miR-155 and miR-375, in T84 tight monolayer cells and that *V*. *cholerae* infected patients express miR-146a and miR-155 in the small intestinal epithelium at acute phase of disease^28^. Intrigued by the finding that OMV-bound VCC does not induce an inflammatory response in tight monolayer cells, we investigated whether OMVs could induce up-regulation of the immunomodulatory microRNAs miR-146a and miR-155^20,21,40,41^ and also miR-375 that is involved in differentiation of intestinal epithelial cells^24,25^. Therefore, samples that had been analysed for cytokine mRNA expression levels were also analysed for expression levels of these three microRNAs. Challenge with crude and density gradient centrifugation purified OMVs, with and without VCC-cargo, for 5 hours caused significant increase in miR-146a levels while only crude, wildtype OMVs caused increased levels of miR-155 (Fig. 3a,b). Soluble VCC did not cause increases in levels of either miR-146a or miR-155 (Fig. 3a,b). These results suggest that OMVs have the capacity to induce increased expression levels of miR-146a whether or not they carry VCC-cargo while non-vesicular components of the bacteria cause increase in levels of miR-155. The magnitude of miR-146a response to OMVs seems to be dependent on dose and/or number of vesicles. Figure 3c shows the results of an experiment in which one wt-OMV-p sample was used for challenge at 100 µg OMV protein (9.2 × 10^6^ vesicles)/monolayer and at 235 µg OMV protein/monolayer, an amount which contained the same amount of vesicles, 21.6 × 10^6^ vesicles, as 100 µg of the *Δvcc*-OMV-p sample used for challenge of parallel monolayers. The response to challenge with 100 µg of the *Δvcc*-OMV-p sample was slightly stronger than the response to challenge with 100 µg wt-OMV-p and almost the same as the response to 235 µg of the wt-OMV-p sample (Fig. 3c). Although the increment was marginal there was a statistically significant increase in the miR-146a response with increasing amount of OMV protein and number of vesicles (*P* < 0.01). Induction of increased miR-146a levels seems to be a quick, relatively long-standing phenomenon since the increase compared to sham-treated controls was the same after 2, 5 and 12 hours (Fig. 3d).

Only OMVs derived from wildtype bacteria induced increase in expression levels of miR-375 (Supplementary Fig. S4a) suggesting that the VCC-cargo is of importance for induction of increased levels of this miRNA.

### *V*. *cholerae* OMVs do not affect expression levels of miR-146a target genes

Since challenge with OMVs caused increased levels of miR-146a the mRNA expression levels of the three miR-146a target genes IRAK1, IRAK2, and TRAF6 were analysed. All three miR-146a targets are expressed in T84 monolayer cells at significant levels (Supplementary Table S1). Challenge with density gradient centrifugation purified OMVs, with or without VCC cargo, did not cause any significant changes in expression levels of mRNA for these genes (Supplementary Fig. S4b–d). The crude wildtype OMV samples caused a significant increase in TRAF6 mRNA levels, supporting the notion of presence of contaminating non-vesicle associated inflammatory components in crude preparations. Interestingly, soluble VCC caused a significant increase in the expression levels of IRAK2 (Supplementary Fig. S4c).

## Discussion

In this study we show for the first time: (1) that *V*. *cholerae* derived OMVs, with and without VCC-cargo, cause significant increase in the expression levels of the immunomodulatory microRNA miR-146a in human intestinal epithelial cells; (2) that OMV-bound VCC has no pro-inflammatory effect on intestinal epithelial cells at doses where soluble VCC has a strong, highly significant pro-inflammatory effect; (3) that soluble VCC causes significant increases in the expression levels of the T lymphocyte attracting chemokine CCL20, the inflammasome cytokine IL-1β and IRAK2, a mediator in the IL-1 signalling pathway, while OMV-bound VCC does not. Furthermore, OMVs of the wildtype bacteria, *i*.*e*. with VCC-cargo, seem to promote epithelial cell differentiation by causing increased levels of the microRNA miR-375 and increase epithelial integrity by decreasing epithelial permeability. These results support the following scenario: *V*. *cholerae* bacteria avoid to trigger a first line of defence reaction at the intestinal epithelial lining of the host by releasing OMVs that reach the epithelium before bacteria themselves and induce increased levels of miR-146a in the epithelial cells, thereby dampening or even abolishing the two innate defence reactions - acute inflammation and inflammasome activation - that are major protective contributions of the epithelial cells upon bacterial attack. Therefore, the bacteria can approach the epithelium without risking strong immune defence reactions against themselves from the host and they can secrete VCC that can exert its toxic actions on eukaryotic cells without raising an inflammatory response of the same magnitude that it would have done if the OMVs had not been released.

That OMVs induce increased levels of miR-146a suggest that they are anti-inflammatory. In line with this notion is the finding that challenge with OMVs, both with and without VCC-cargo, did not cause increases in levels of any of the inflammatory cytokines analysed, *i*.*e*. IL-8, TNF-α, CCL20, IL-1β, and IL-18. The finding that OMVs without VCC-cargo, in two cases of three, even suppressed the baseline levels of mRNAs for CCL20 and the inflammasome cytokine IL-18 further strengthen the notion that OMVs are anti-inflammatory. Down-regulation of IL-18 suggests reduced capacity to activate intraepithelial lymphocytes that normally execute immune protection at the epithelial lining^39,42,43^. Thus, *in vivo* exposure of the epithelium to OMVs might reduce the defence capacity of the two cell-types of intestinal epithelium that together build a protective barrier towards the gut lumen, *i*.*e*. the epithelial cells and the intraepithelial lymphocytes. In line with this hypothesis, we observed that patients with *V*. *cholerae* O1 infection at the acute stage of disease expressed the miR-146a and miR-155 in the small intestinal epithelium and had only weak cytokine responses in their intestinal mucosa with no increases in interferon-γ or IL-17A that are typically produced by activated intraepithelial lymphocytes^28,32,44^.

miR-146a targets multiple key mediators of the NF-κB pathway^40^. No decrease in the expression levels of the miR-146a target genes IRAK1, IRAK2, and TRAF6 were observed after OMV-challenge. In contrast, soluble VCC caused significant increase of IRAK2. Thus, it seems that in intestinal epithelial cells miR-146a prevents up-regulation rather than cause down-regulation of these signalling molecules.

Together, our previous^9^ and present studies on epithelial responses to challenge with soluble VCC suggest that VCC alone can elicit a full-blown immune response at the site of infection in the intestinal mucosa. The fact that it caused increased levels of IRAK2 suggests that VCC can indeed cause severe inflammation, since studies in mice showed that IRAK2 is associated with sustained inflammation and LPS-induced shock^23^. Thus, it seems that VCC is an important virulence factor of *V*. *cholerae*, in particular of the non-CT/TCP producing NOVC strains. However, although OMVs of the wildtype NOVC *V*. *cholerae* V:5/04 strain used here carry VCC (this study and ref.^17^), they did not cause inflammatory responses. This divergence in action between soluble and OMV-bound VCC could be due to that the OMV-bound and the free, soluble VCC exist in different structural forms, a notion supported by the anti-VCC immunoblot analysis.

Two OMV induced phenomena were associated to presence of VCC on the OMVs since they occurred after challenge with OMVs from wildtype *V*. *cholerae* but not after challenge with OMVs from the VCC deletion mutant: (1) tightening of the monolayer and (2) increase in miR-375 levels. The finding that OMV-bound VCC caused significant increase in TER, while soluble VCC caused a marked decrease, suggests that the two forms affect tight junction structures differently. That OMVs can cause increase in TER by reinforcement of tight junctions has been reported for a probiotic *E*. *coli* strain^45^. Challenge with OMV-bound VCC caused increases in miR-375 but miR-375 was not detected in the small intestinal mucosa of patients with *V*. *cholerae* infection^28^. Thus, increases in miR-375 levels seem to be a feature of the polarized tight monolayer *in vitro* model. miR-375 is involved in directing differentiation of epithelial cells into goblet cells and/or neuroendocrine cells^24,25^, suggesting that the OMV-bound VCC triggers differentiation in the monolayer cells. The discrepancy between the situation in the patients and the *in vitro* studies could be explained by the fact that the monolayers are established from T84 cells that are of colonic origin while the studies in *V*. *cholerae* infected patients was performed on small intestinal biopsies.

Presence or absence of VCC was not the only observed difference between OMVs of the wildtype and the VCC deletion mutant. Thus, OMVs of the VCC deletion mutant had higher numbers of vesicles per mg protein and a more uniform size than OMVs of the wildtype. It is not clear to what extent these properties influence the outcome of challenge with OMVs without VCC-cargo. Experiments in which the effects of OMVs with and without VCC-cargo were compared after adjustment to the same amount of protein or to the same numbers of vesicles for challenge, respectively, did not answer this question. However, it seems that tightening of the epithelium caused by OMVs with VCC-cargo occurs preferentially at challenge with lower numbers of OMVs, suggesting that OMV mediated decrease of epithelial permeability occurs early in *V*. *cholerae* onslaught in the patients. It is not known how OMVs interact with the tight monolayers, if they stay at the cell surface or enter into the cells. Several mechanisms of OMV entry into eukaryotic cells have been reported including different routes of endocytosis and even membrane fusion^46^. If any of these routes of entry apply to epithelial cells in tight monolayers or intestinal epithelium *in vivo* has not been studied. It can be speculated that the size of the OMV directs them towards a specific uptake route.

Over all, VCC soluble protein and OMVs, with or without VCC cargo, seem to have opposite effects. VCC raises strong inflammatory responses while OMVs down-regulate inflammation. Given the facts that one sees virtually no T cell response in the small intestinal mucosa of *V*. *cholerae* infected patients at the acute stage of disease and that the inflammatory response is weak^28^ it appears that OMVs and the bacteria themselves counteract the inflammatory effects of VCC by inducing miR-146a and miR-155, respectively, in the epithelial cells. By releasing factors that harbour both inflammation-inducing and inflammation-suppressing capabilities, *V*. *cholerae* bacteria can manipulate the host immune responses without requiring direct contact with host cells at the epithelial lining. Through this way of evading the immune system, the bacteria can thrive within the host and may even cause severe disease, as observed in patients infected with *V*. *cholerae* O1-, O139-, as well as NOVC strains.

## Supplementary information

Supplementary info

## Data Availability

All relevant data are within the manuscript, Supplementary Table S1 and Fig. S1 through S4.
