# Supplementary material for: Vibrio cholerae derived outer membrane vesicles modulate the inflammatory response of human intestinal epithelial cells by inducing microRNA-146a
Source: Sci Rep. 2019 May 10;9:7212. doi: 10.1038/s41598-019-43691-9 (PMC6510749; doi:10.1038/s41598-019-43691-9)
Supplement: Supplementary file 1 — Supplementary info [file 41598_2019_43691_MOESM1_ESM.pdf]

***Vibrio cholerae* derived outer membrane vesicles modulate the inflammatory response of human intestinal epithelial cells by inducing microRNA-146a.** Aziz Bitar, Kyaw Min Aung, Sun Nyunt Wai, Marie-Louise Hammarström

Supplementary Figure S1a

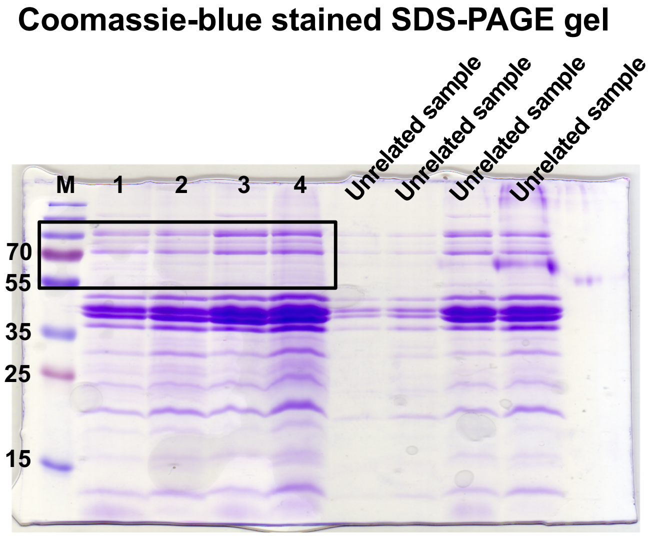

The box indicates the area displayed in Figure 1c

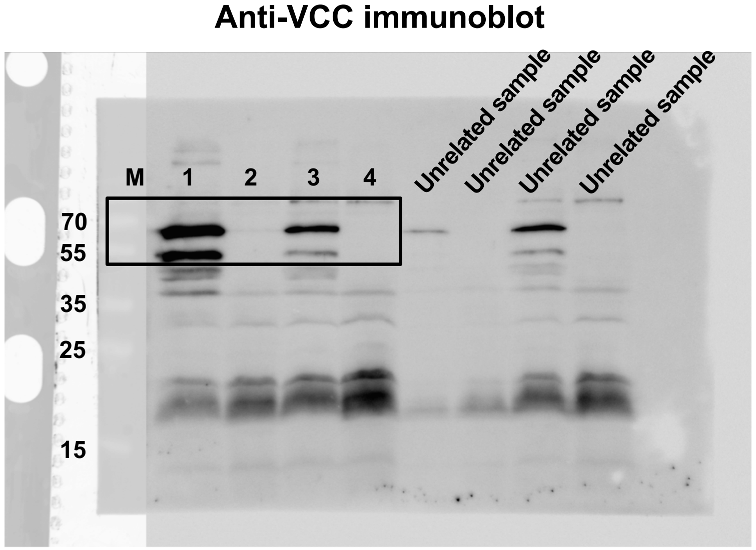

The box indicates the area displayed in Figure 1d

Figure legend: Coomassie-brilliant-blue-stained SDS-PAGE gel of one of the wildtype outer membrane vesicle (wt-OMV) samples and one of the *Δvcc*-OMV samples from *Vibrio cholerae* strain V:5/04 utilized in the study (left) and anti-VCC immunoblot analysis of the same gel (right). Lane 1, crude OMV preparation from the wildtype (wt-OMV-c); lane 2, crude OMV preparation of the VCC deletion mutant (*Δvcc*-OMV-c); lane 3, OMVs from the wildtype further purified by density gradient centrifugation (wt-OMV-p); and lane 4, OMVs from the VCC deletion mutant further purified by gradient centrifugation (*Δvcc*-OMV-p). Molecular weight markers were run in the lane marked M. Other lanes are unrelated samples. Sizes of molecular weight markers are given in kDa to the left of the gels.

Vibrio cholerae **derived outer membrane vesicles modulate the inflammatory response of human intestinal epithelial cells by inducing microRNA-146a.** Aziz Bitar, Kyaw Min Aung, Sun Nyunt Wai, Marie-Louise Hammarström

Supplementary Figure S1b

A. Image of anti-VCC immunoblot membrane taken under a LAS 4000 imager using a precision exposure type program

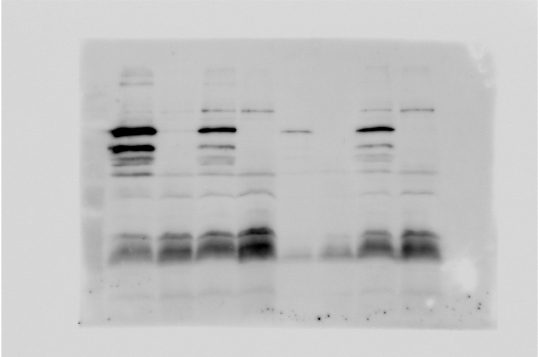

B. Image of molecular weight markers on the anti-VCC immunoblot membrane taken under a LAS 4000 Imager using an *in vivo* exposure type program.

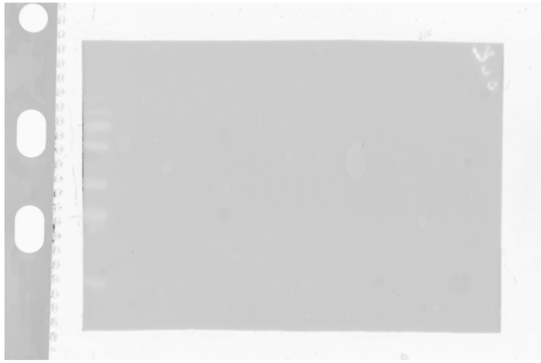

Merge of images A and B giving the exact positions of the bands in the anti-VCC immunoblot. The box indicates the area displayed in Figure 1d.

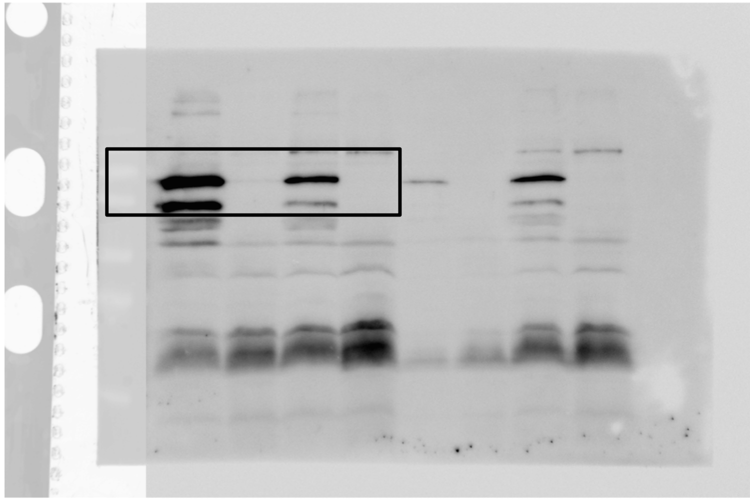

***Vibrio cholerae* derived outer membrane vesicles modulate the inflammatory response of human intestinal epithelial cells by inducing microRNA-146a.** Aziz Bitar, Kyaw Min Aung, Sun Nyunt Wai, Marie-Louise Hammarström

Supplementary Figure S2a

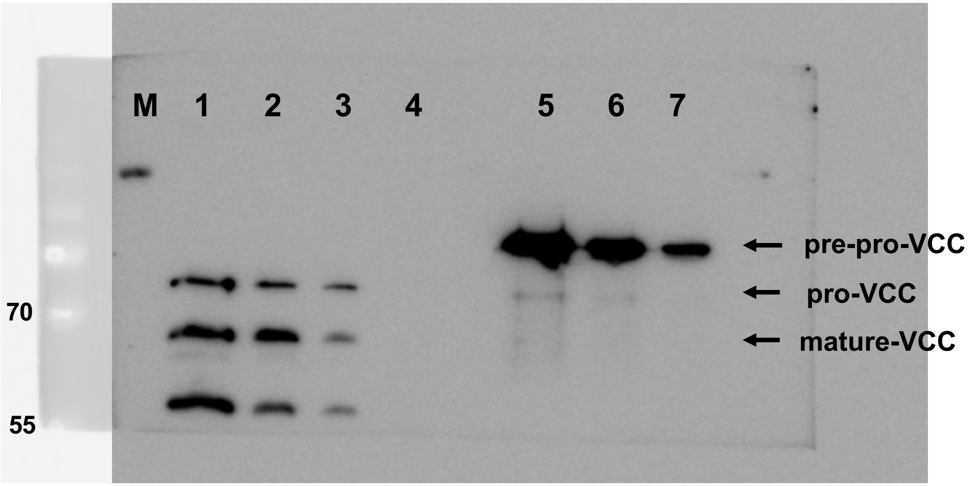

Figure legend: Anti-VCC immunoblot analysis of outer membrane vesicle (OMV) samples purified by density gradient centrifugation from wildtype *Vibrio cholerae* strain V:5/04 (wt-OMV-p; lines 1–3), *Vibrio cholerae* strain V:5/04 deleted of the gene for VCC (*Δvcc*-OMV-p; lane 4) and soluble, recombinant VCC (lanes 5–7). Amount of protein added per slot was 15.4, 7.7, 3.1, and 1.0 µg for lanes 1–4 and 15.0, 7.5 and 6.0 ng for lanes 5–7. The pre-pro-VCC with molecular weight of 82 kDa, the pro-VCC with molecular weight 79 kDa and the mature-VCC with molecular weight 68 kDa are indicated with arrows. Molecular weight markers were run in the lane marked M. Sizes of molecular weight markers are given in kDa to the left of the gel.

***Vibrio cholerae* derived outer membrane vesicles modulate the inflammatory response of human intestinal epithelial cells by inducing microRNA-146a.** Aziz Bitar, Kyaw Min Aung, Sun Nyunt Wai, Marie-Louise Hammarström

Supplementary Figure S2b

i. Image of anti-VCC immunoblot membrane taken under a LAS 4000 imager using a precision exposure type program.

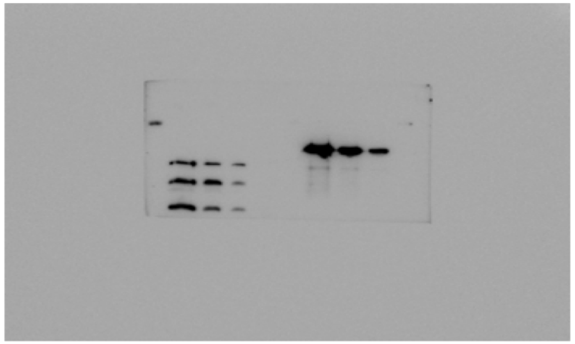

ii. Image of molecular weight markers on the anti-VCC immunoblot membrane taken under a LAS 4000 Imager using an *in vivo* exposure type program.

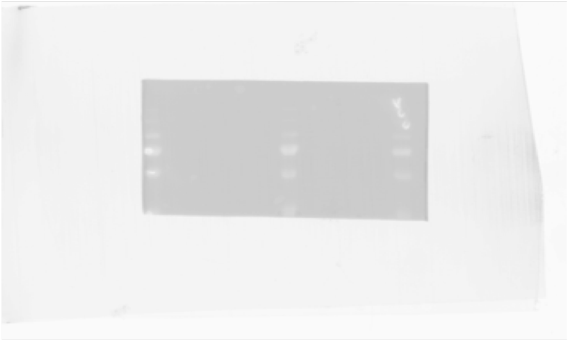

Merge of images i and ii giving the exact positions of the bands in the anti-VCC immunoblot.

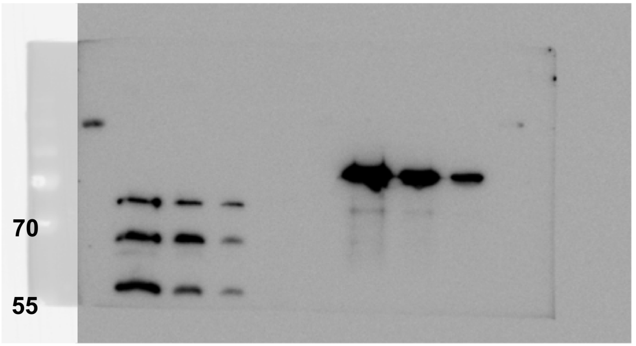

Vibrio cholerae **derived outer membrane vesicles modulate the inflammatory response of human intestinal epithelial cells by inducing microRNA-146a.** Aziz Bitar, Kyaw Min Aung, Sun Nyunt Wai, Marie-Louise Hammarström

Supplementary Figure S2c

Quantification of density of bands obtained from anti-VCC immunoblot analysis using the Quantity One 1-D Analysis software

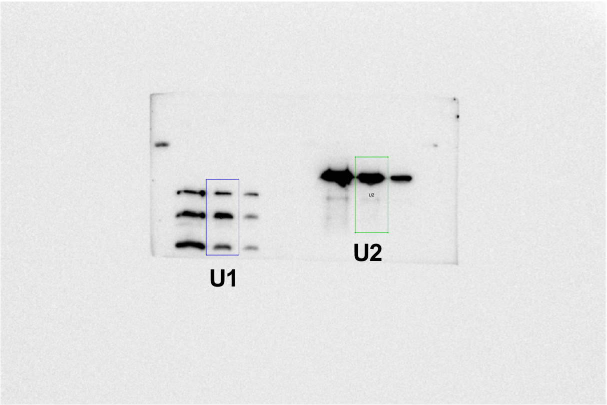

Index	Name	% Adj. Vol.	Density INT/mm2
1	U1	48.94	882.6181840
2	U2	51.06	920.6916724

U1: total 7.7 µg of OMVs from wildtype *Vibrio cholerae* strain V:5/04

U2: total 7.5 ng of soluble, recombinant VCC

Supplementary Figure S2d

Immunoblot densitometry analysis of OMV-associated VCC from wildtype *Vibrio cholerae* strain V:5/04 and purified recombinant VCC

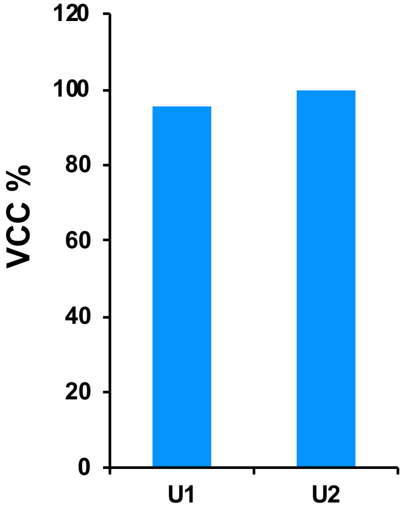

U1: Percentage of associated VCC in total 7.7 µg of wildtype *Vibrio cholerae* strain V:5/04 OMVs (95.8%)

U2: Percentage of total soluble, recombinant VCC in 7.5 ng (100%)

Vibrio cholerae **derived outer membrane vesicles modulate the inflammatory response of human intestinal epithelial cells by inducing microRNA-146a.** Aziz Bitar, Kyaw Min Aung, Sun Nyunt Wai, Marie-Louise Hammarström

Supplementary Figure S3

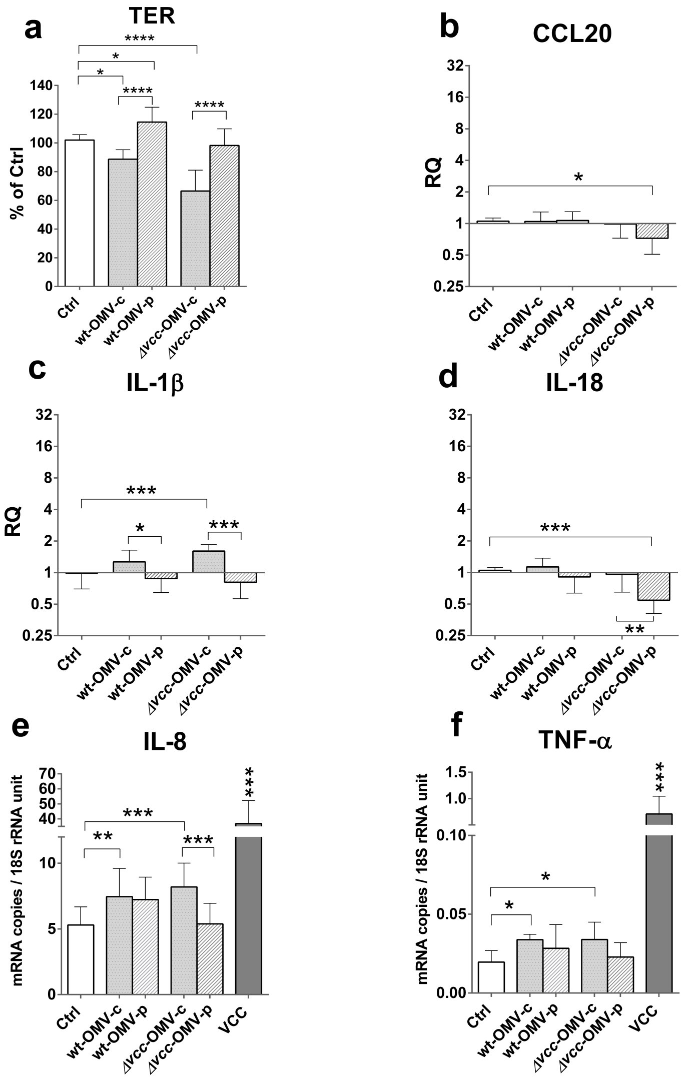

Figure legend. Polarized tight monolayers of T84 cells were challenged at the apical side with OMVs derived from the *V. cholerae* strain V:5/04 and its VCC deletion mutant before (wt-OMV-c and *Δvcc*-OMV-c; dotted bars) and after (wt-OMV-p and *Δvcc*-OMV-p; striped bars) density gradient centrifugation. **(a)** changes in TER and **(b)** - **(f)** changes in levels of mRNA for CCL20 (**b**), IL-1β (**c**), IL-18 (**d**) IL-8 (**e**) and TNF-α (**f**) in monolayers challenged with wildtype, crude OMVs (wt-OMV-c; n=8), density gradient centrifugation purified wildtype OMVs (wt-OMV-p; n=8), VCC deletion mutant crude OMVs (Δ*vcc*-OMV-c; n=8), density gradient centrifugation purified VCC deletion mutant OMVs (Δ*vcc*-OMV-p; n=8) and sham-treated monolayers (Ctrl, open bars; n=8 in **a**–**d** and 12 **e**–**f**). (**e**) **-** (**f**) also show the results from monolayers challenged with soluble VCC (VCC, filled bars; n=4). Results in (**a**) are given as % of controls (% of Ctrl) for individual monolayers relative to the mean %TER of sham-treated control monolayers in the respective experiment. Results in (**b**) - (**d**) are given as relative quantity (RQ) compared to the median level of sham-treated monolayers. Results in (**e**) **-** (**f**) are given as mRNA copies/18S rRNA unit. Results are from two independent experiments in which two batches of wt-OMVs and two batches of Δ*vcc*-OMVs were investigated for effects of challenge with paired samples of crude and density gradient centrifugation purified OMVs. Challenges were done with 100 µg total OMV protein/monolayer and 80 ng VCC/monolayer for 5 hours. Bars indicate mean + 1 SD. Statistically significant differences are shown; **** P-value <0.0001, *** P-value <0.001, ** P-value <0.01; * P-value <0.05.

Vibrio cholerae derived outer membrane vesicles modulate the inflammatory response of human intestinal epithelial cells by inducing microRNA-146a. Aziz Bitar, Kyaw Min Aung, Sun Nyunt Wai, Marie-Louise Hammarström

Supplementary Figure S4

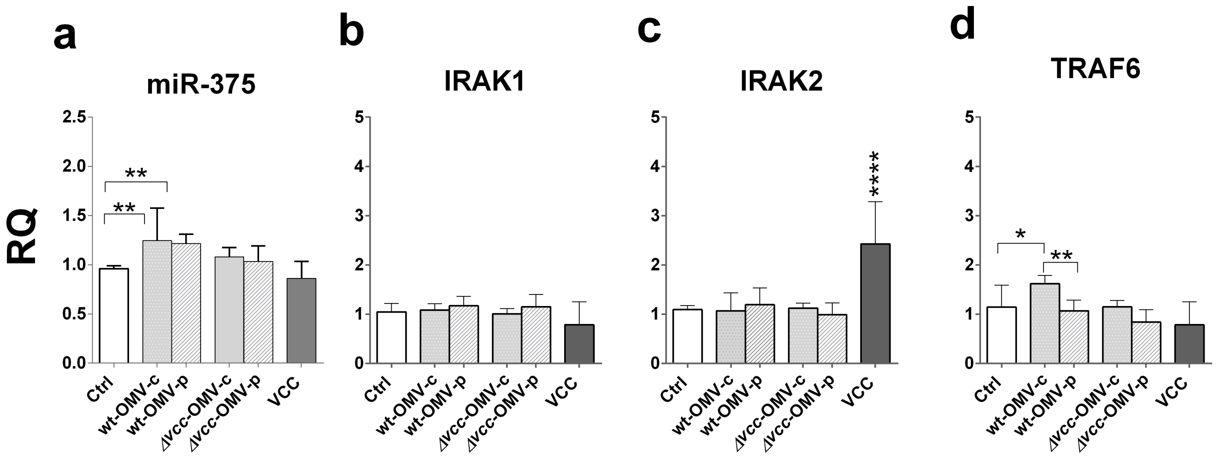

Figure legend. The same RNA samples that were analysed for TER and cytokine mRNA expression levels with results shown in supplementary Figure S3 and monolayers challenged with 80 ng soluble VCC were also analysed for miR-375 (a), IRAK1 mRNA (b), IRAK2 mRNA (c) and TRAF6 mRNA (d) expression levels. Amounts of miR-375 were determined by real-time qRT-PCR and normalized to the content of RNU48 in the sample. Amounts of IRAK1, IRAK2, and TRAF6 mRNA were determined by a real-time qRT-PCR and normalized to the content of 18S rRNA in the sample. Results are expressed as relative quantity (RQ) relative to the median of sham-treated tight monolayers incubated in parallel with monolayers challenged with OMVs. Bars indicate mean RQ + 1 SD. Statistically significant differences are shown, **** _P_-value <0.0001, *** _P_-value <0.001, ** _P_-value <0.01 and * _P_-value <0.05.

***Vibrio cholerae* derived outer membrane vesicles modulate the inflammatory response of human intestinal epithelial cells by inducing microRNA-146a**

Aziz Bitar, Kyaw Min Aung, Sun Nyunt Wai, Marie-Louise Hammarström

Supplementary Table S1. Expression levels of cytokine mRNAs, microRNAs and microRNA miR-146a target gene mRNAs in sham-treated T84 polarized tight monolayer cells.

**mRNA or microRNA species**	**Δct-value***	**n°**
Cytokine mRNAs		
IL-8	3.9 (2.4-4.3)	12
TNF-*α*	8.8 (8.1-9.4)	12
CCL20	6.4 (5.7-6.9)	16
IL1-β	6.4 (5.9-6.8)	16
IL-18	5.6 (4.3-5.9)	16
microRNAs		
miR-146a	9.7 (9.2-10.3)	16
miR-155	3.2 (2.9-3.4)	12
miR-375	3.8 (3.1-4.5)	12
miR-146a target gene mRNAs		
IRAK1	6.0 (5.8-6.3)	12
IRAK2	5.9 (5.5-6.1)	12
TRAF6	5.3 (4.1-6.1)	12

* Δct-value for mRNAs: Ct-value for the mRNA species of interest minus ct-value of 18S rRNA in a 1:1000 dilution of the same sample.

Δct-value for microRNAs: Ct-value for the microRNA species of interest minus ct-value of RNU48 in the same sample.

Results are given as median and interquartile range from the 25th to the 75th percentile.

° n = number of sham-treated control monolayers analysed.
